# Maintenance of genetic diversity in cyclic populations—a longitudinal analysis in *Myodes glareolus*

**DOI:** 10.1002/ece3.277

**Published:** 2012-07

**Authors:** Kaisa Rikalainen, Jouni Aspi, Juan A Galarza, Esa Koskela, Tapio Mappes

**Affiliations:** 1 Department of Biological and Environmental Science, University of JyväskyläP.O. Box 35, FI-40014 Jyväskylä, Finland; 2 Department of Biology, University of OuluP.O. Box 3000, FI-90014 Oulu, Finland; 3 Department of Biological and Environmental Science, Centre of Excellence in Biological Interactions, University of JyväskyläP.O. Box 35, FI-40014 Jyväskylä, Finland

**Keywords:** Allelic richness, effective population size, genetic diversity, *Myodes glareolus*, private alleles, rodent cycles

## Abstract

Conspicuous cyclic changes in population density characterize many populations of small northern rodents. The extreme crashes in individual number are expected to reduce the amount of genetic variation within a population during the crash phases of the population cycle. By long-term monitoring of a bank vole (*Myodes glareolus*) population, we show that despite the substantial and repetitive crashes in the population size, high heterozygosity is maintained throughout the population cycle. The striking population density fluctuation in fact only slightly reduced the allelic richness of the population during the crash phases. Effective population sizes of vole populations remained also relatively high even during the crash phases. We further evaluated potential mechanisms contributing to the genetic diversity of the population and found that the peak phases are characterized by both a change in spatial pattern of individuals and a rapid accession of new alleles probably due to migration. We propose that these events act together in maintaining the high genetic diversity within cyclical populations.

## Introduction

Since the pioneering work of Elton (1924) on lemming populations, the density oscillation in numbers of small mammals has been a subject of a vast amount of study in population ecology (reviewed by, e.g., Krebs and Myers 1974; Hansson and Henttonen 1988; Stenseth 1999). Drastic examples of this population density oscillation are the northern vole populations, which reach their maximum approximately every third or fourth year with more than 10-fold increase in individual numbers compared to the low population density (Krebs 1996). After reaching peak density, the population is almost wiped out within a few months. However, true extirpation is not the case, since the population recovers effectively to reach its peak density again after three to four years. While explaining the causes of the population density oscillation (see e.g., Krebs et al. 1995, 1996; Boonstra et al. 1998; Sinclair et al. 2003; Korpimäki et al. 2005; Lambin et al. 2006; Smith et al. 2006, 2008; Massey et al. 2008), the research of its effects on the genetic diversity and composition of a population is only beginning to accumulate (Ehrich and Jorde 2005; Vuorinen and Eskelinen 2005; Berthier et al. 2006; Ehrich et al. 2009).

The striking population density oscillation has the potential to reduce genetic diversity and generate spatial genetic structure in the population during crash phases. Loss of genetic diversity predisposes the population to genetic erosion, which is most obviously seen in the populations that have encountered bottleneck (Nei et al. 1975; Saccheri and Hanski 2006). Nevertheless, despite the strong oscillation in population density and the possible threat of repetitive bottlenecks, high heterozygosity has been frequently observed among several vole populations (see e.g., Plante et al. 1989; Berthier et al. 2005, 2006; Ehrich and Jorde 2005; Redeker et al. 2006; Ehrich et al. 2009). The heterozygosity in cyclic populations may be maintained by various processes of individual behavior and selection acting on the population. The processes suggested previously include differences in dispersal pattern (migration) throughout the density cycle, short duration of the crash phase accompanied with weak genetic drift, and a rapid accumulation of new alleles through mutation or immigration (Berthier et al. 2005; Ehrich and Jorde 2005; Ehrich et al. 2009). Moreover, inbreeding avoidance mechanisms, such as spacing behavior and mating pattern (Pusey and Wolf 1996; Perrin and Goudet 2001) may increase the genetic diversity within a population and influence its spatial genetic structure. Temporal environmental heterogeneity (i.e., temporally varying selection, e.g., Roff 1997) may also favor different alleles (and further different genotypes, Chitty 1967) during different phases of the population cycle. Long-term genetic monitoring on populations is vital in order to establish the changes in population's genetic composition and examine the mechanisms that can contribute to the maintenance of high genetic diversity throughout the density cycle. Thus far, the genetic research within cyclical rodent populations on this topic has either concentrated on a single phase of the density cycle or has had a rather explorative quality (Berthier et al. 2005, 2006; Ehrich and Jorde 2005; Redeker et al. 2006; Ehrich et al. 2009).

In order to test the hypothesis that high genetic variability is maintained in the population despite the repetitive population crashes, we performed a time-series study of a bank vole (*Myodes glareolus*) population with a dynamic demography (Kallio et al. 2009). We monitored the population for eight consecutive years; a period of three cycles of population density peaks and crashes and compared the peak and the crash phases in terms of genetic diversity, effective population size, bottleneck signature, private alleles, selection, and spatial genetic structure. We expected to find some signs of genetic depletion, such signals of population bottlenecks, distinctive population structure, and low effective population size during the crashes. We also aimed to trace the possible mechanisms, such as spatial distribution and selection, which could maintain high genetic variability in cyclic populations.

## Methods

### Study species, population size, and DNA sampling

**Figure 1:**
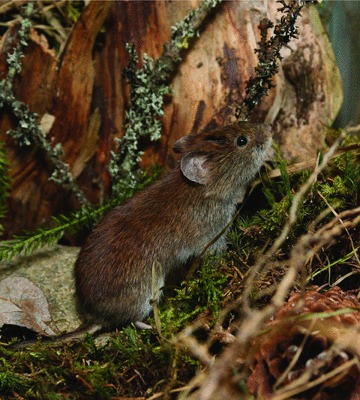
The study species, the bank vole (*Myodes glareolus*). Photograph by Heikki Helle.

The bank vole (*M. glareolus*, Fig. 1) is a common northern European rodent (Stenseth 1985), which inhabits forests and fields, feeding on plants, seeds, and fungi (Hansson 1985). The life-history pattern of the bank vole is characterized by a short life span, young age of maturation, high fecundity, short gestation period, and up to four breeding events within the same reproductive season. There is also high phenotypic and genetic variation in these life-history traits (see e.g., Koivula et al. 2003; Mappes and Koskela 2004; Mappes et al. 2008; Schroderus et al. 2012).

**Figure 2:**
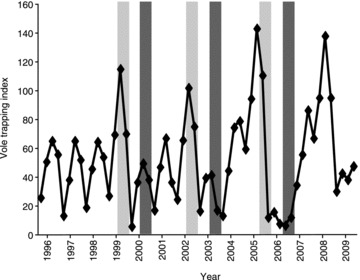
The trapping index of bank voles during 1996–2009 (trapping index = captured individuals/100 trap nights, monthly data are interpolated from the trappings carried out four times per year; trappings are indicated with diamonds). The six analyzed cycle phases are indicated with light (peak years) and dark (crash years) bars.

In this study, we followed the bank vole population in Central Finland (62°37′N, 26°20′E) for eight consecutive years (1999–2006), during which the population size oscillated in three successive cycles (Fig. 2). For monitoring the cycle, the population was trapped four times per year: in May (early breeding season), July (middle of the breeding season), August (late breeding season), and late October–early November (after the breeding season). Altogether 20 constant trapping sites were distributed over an area of approximately 100 km^2^ and each of them contained four Ugglan Special multiple-capture live traps (Grahnab, Hillerstorp, Sweden) that were situated at each corner of a 15-m square. The mean distance between any two trapping sites was 4.3 km. The trapping sites were located in a coniferous forest dominated by Scots pine (*Pinus sylvestris*), Norway spruce (*Picea abies*), and various shrubs (e.g., *Calluna* sp., *Vaccinium* spp.). Before each trapping session, the traps were prebaited with sunflower (*Helianthus annuus*) seeds for two days and then set for two consecutive days and nights and checked daily. To represent the size of our study population, we calculated the trapping index (Fig. 2) as the number of captured individuals per 100 trap nights, where monthly trapping data are interpolated from the trappings carried out four times per year. Bank voles have very good trappability (see e.g., Kallio et al. 2007) and so this index gives a very accurate estimation of the population density over time. The density cycle phase categorization was further established based on autocorrelation analysis according to Kallio et al. (2009).

The individuals genotyped for the present study were trapped during the third and fourth trapping of the year (late and after breeding season) at three peak (years 1999, 2002, and 2005) and three crash (years 2000, 2003, and 2006) density phases of the cycle. We used one adult individual (born in the sampled year) per sampling location and per study year (i.e., 20–22 voles per each study year, 130 in total, see Table A1), and their sex and weight were recorded. None of the samples varied from an equal sex ratio. Tissue samples were taken by ear clipping and stored individually at –80°.

### Microsatellite analysis

DNA was extracted from the tissue samples (Kingfisher magnetic particle processor, Thermo Fisher Scientific, Waltham, U.S.A.) and the individuals were genotyped at 28 microsatellite loci as described in Rikalainen et al. (2008). The microsatellites used were Cg2D8, Cg2F2, Cg3F12, Cg3E12, Cg1F11, Cg5G6, Cg6D10, Cg8A5, Cg1E6, Cg17C9, Cg17A7, Cg16E2, Cg15F5, Cg13G2, Cg12H10, Cg10F6, Cg10D11, Cg12E6, Cg13B8, Cg13C12, Cg13F9, Cg17E9, Cg12B9, Cg1A8, Cg2C5, Cg3D12, Cg16H5, and Cg14A5, and the PCR amplification conditions were carried out as described in Rikalainen et al. (2008). The amplified fragments were detected with an ABI Prism 3100xl (Applied Biosystems, Carlsbad, California, U.S.A.) and scored using genemapper v3.7 software (Applied Biosystems). Any sample failing to produce clear signals was reamplified under the same conditions.

The software micro-checker v2.2.3 (Van Oosterhout et al. 2004) was used to identify possible errors (null alleles, large allele drop out, stuttering, and typographic errors). The statistical analysis suggested null alleles at five loci (Cg1A8 [null allele frequency *F*_i_ = 0.296], Cg2C5 [*F*_i_ = 0.134], Cg3D12 [*F*_i_ = 0.241], Cg16H5 [*F*_i_ = 0.106], and Cg14A5 [*F*_i_ = 0.254]), which were omitted from further analyses.

### Genetic diversity, population's temporal differentiation, and demographic changes

In order to investigate whether the population maintains its genetic diversity throughout the density cycle, we conducted several population genetic assays and compared the opposing cycle phases (three peaks and three crashes). First, we tested all the feasible 23 microsatellite loci for linkage disequilibrium by Fisher's exact test implemented in the software genepop on the web (Raymond and Rousset 1995) as well as deviations from Hardy–Weinberg expectations using the software fstat v2.9.3.2 (Goudet 1995). To see whether there are differences in genetic diversity and inbreeding status on individuals between the crash and the peak phases, we calculated heterozygosity levels and estimates for allelic richness (i.e., a measure of the number of alleles independent of sample size), and inbreeding coefficient (*F*_IS_) for each analyzed year by using fstat. To evaluate whether the genetic population structure changes during the three population cycles, we computed pairwise *F*_ST_ (Weir and Cockerham 1984) values for each analyzed pair of years. We used a false discovery rate (FDR) approach (Benjamini and Hochberg 1995) to correct the possible type I errors in multiple testing in both the significance of deviations from Hardy–Weinberg proportions and the pairwise *F*_ST_ values.

We wanted to see to what extent the peak and the crash phases differed from each other in terms of genetic variability measured as heterozygosity levels, allelic richness, inbreeding status (*F*_IS_), and genetic structure (*F*_ST_). We compared the peak and crash phases by using the “Comparison among groups of samples”—option in fstat (group 1: peak years 1999, 2002, and 2005, and group 2: crash years 2000, 2003, and 2006). Similarly, to detect if significant changes had occurred in the population's genetic composition during the study period, we performed a Mantel test (see e.g., Manly 1997) in tfpga v1.3 (Miller 1997) between the *f*_ST_ and the temporal distance matrices. Significance was attained by means of 10,000 permutations, which is a realistic minimum for estimating a significance level of approximately 0.01 (Manly 1997).

To detect the possible signs of genetic bottlenecks within the population, we used the software bottleneck v1.2 (Cornuet and Luikart 1996) for computing the heterozygosity excess (i.e., whether the heterozygosity computed from a sample of genes is larger than the heterozygosity expected from the number of alleles found in the sample of a constant size population) for each study year. We performed the analyses using the two-phased mutation model, Wilcoxon sign-rank test with 95% of single-step mutations, and variance among multiple steps of 16 as recommended for microsatellite loci (Piry et al. 1999). We also obtained the Garza–Williamson index (the number of alleles divided by the allelic range that is expected to be low in bottlenecked population, Garza and Williamson 2001) for all phase points using arlequin v3.1 (Excoffier et al. 2005).

To further infer the population history and changes in population size over time, we used a coalescent-based approximate Bayesian computation software diyabc (Cornuet et al. 2008; see also Robert et al. 2011). The coalescence analyses are population genetic models that attempt to look backwards in time to examine genealogy of alleles until the most recent common ancestor is reached. We defined two models, one assuming constant population size and the other allowing the population size to change during the analyzed period. Prior uniform distributions were set for *N*e (lower limit 100 and upper limit 100,000), mean mutation rate *µ* (lower limit 1 × 10^–5^ and upper limit 1 × 10^–3^), and a gamma distribution for locus *P* (lower limit 1 × 10^–5^ and upper limit 1 × 10^–2^, with shape 2.0). The stepwise mutation model was used and 1 million replicate runs were preformed to generate the reference tables.

### Spatial distribution, new alleles, and sign of selection

In order to elucidate the possible mechanisms contributing to the maintenance of high genetic diversity within the population, we first analyzed the spatial genetic structure of the population in two ways. We were interested to see whether the population displays a distinctively patchy population structure during the crashes and whether there are differences in isolation-by-distance at opposing cycle phases. These two events could signal differences in spacing pattern of individuals during the population cycle.

First, we performed a probabilistic Bayesian clustering test with the software structure (“admixture model,” 10 repeats of 1,000,000 Markov chain Monte Carlo iterations +300,000 as a burn-in [Pritchard et al. 2000]) to infer how many breeding units are the most appropriate for interpreting the data without prior information about the number of locations and individual's origin. Large number of separate breeding units can be interpreted to a patchy population structure. We conducted the analysis for each study year separately (i.e., three peaks and three crashes) to discern the differences between the opposing cycle phases, and also for data combined over all study years.

Second, we conducted spatial autocorrelation analysis using the software spagedi (Hardy and Vekemans 2002) to evaluate the relationship between the kinship coefficient of the individuals and geographical distance. The autocorrelation between individuals’ relatedness (kinship coefficient) and their geographical distance can refer to individual movement through their distribution in space, and differences in this correlation between cycle phases may indicate periodic changes in migration and dispersal. In order to compare the opposing cycle phases in terms of spatial distribution, we categorized the data into two groups according to the cycle phase and used the Loiselle et al. (1995) estimator of kinship coefficient, which is especially suitable in cases with low-frequency alleles present (Hardy and Vekemans 2002). Since there is no general consensus regarding the way to generate distance classes, we used the equal frequency method where the software creates uneven distance classes that contain an equal number of samples among them (Esqudero et al. 2003). Moreover, we analyzed the spatial genetic structure of female and male individuals separately for the whole dataset and also for crash and peak phases separately.

New genetic material that is accumulated to the population can contribute to the allelic variety and the maintenance of genetic diversity within a population. In order to see whether new alleles are frequently or even cyclically introduced to the population, we calculated the number of private alleles (i.e., an allele unique to one study year) at each locus using the software arlequin (Excoffier et al. 2005) and compared the number of private alleles at each locus between the peak and crash phases.

To test if one or more of the analyzed loci were linked to a particular population cycle phase and would therefore sign for temporal heterogeneity favoring different alleles in different phases of the population cycle, we performed a test of analysis of molecular variance (AMOVA), using the software arlequin v3.1 (Excoffier et al. 2005). We first divided the analyzed years into two groups according to the population cycle phase and performed the locus-by-locus analysis. We used FDR approach (Benjamini and Hochberg 1995) to correct the possible type I errors in multiple testing of the significance of AMOVA.

## Results

### Genetic diversity, temporal differentiation, and demographic changes

In our sample, the individuals’ mean weight at crash phases was 15.76 g (SD = 3.71) and at peak phases 15.97 g (SD = 3.00). The annual mean of trapping index varied from 16.5 individuals per 100 trap nights (at the crash year 2006) to 163 individuals per 100 trap nights (at the peak year 2005), and the population size was lowered by 54% (transition 1999–2000), 57% (transition 2002–2003), and 90% (transition 2005–2006) at the transitions from peak year to crash year. This indicates that the study population goes through substantial and repetitive crashes in population size.

We found some genetic linkage between loci (19 out of 120 locus pairs, Fisher exact test), but none of the locus pairs appeared to be constantly at linkage disequilibrium during the analyzed phase points. However, we noted that most of the disequilibrium was evident at one crash year (year 2003, 14 out of 19 locus pairs), probably because recent population size reductions typically increase the linkage disequilibrium between loci (McVean 2002).

**Figure 3:**
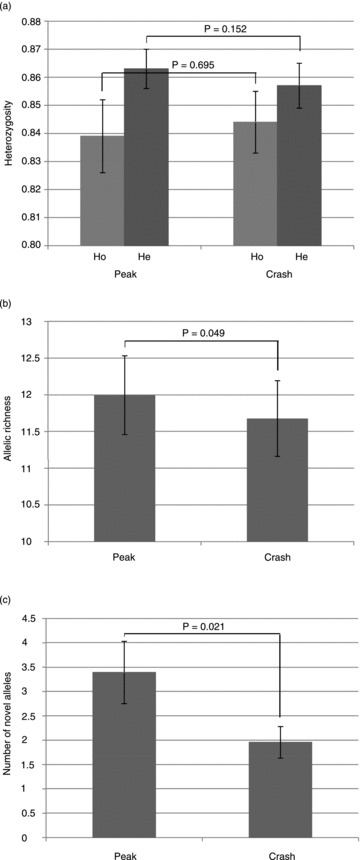
Differences in (a) observed (*Ho*) and expected (*He*) heterozygosity, (b) allelic richness, and (c) number of novel alleles between the crash and the peak phases of the bank vole population (mean ± SE).

All the loci used in our analyses were highly polymorphic, having allele number ranging from 5 to 31 per locus per cycle phase point (see Table A1) with an average of 12.65–14.26. Four loci (Cg2D8, Cg8A5, Cg10D11, and Cg12B9) showed deviation from Hardy–Weinberg expectations (randomization test with FDR), but none of these loci was repeatedly at disequilibrium at the analyzed cycle phase points (exact Hardy–Weinberg (HW) test). The observed (*Ho*) and expected (*He*) heterozygosities (Tables 1 and A1) were high and constant over the analyzed period and there were no statistical differences between crash (mean *Ho* = 0.844, *He* = 0.857) and peak (mean *Ho* = 0.839, *He* = 0.863) cycle phases (Fig. 3a, randomization test, *P_H_*_o_ = 0.695, *P_H_*_e_ = 0.152). We did not find any direct evidence for inbreeding (*f*_IS_, Table 1) at any of the study years (randomization test, all *P* > adjusted nominal level of 0.00036). Moreover, there were no statistical differences between crashes (mean *F*_IS_ = 0.040) and peaks (mean *F*_IS_ = 0.051) (randomization test, *P* = 0.354).

**Table 1:** Population genetic characteristics of the bank vole population during three population density peak and crash phases (three population cycles): expected (*He*) and observed (*Ho*) heterozygosity, effective population size (*Ne*), number of alleles (*A*), allelic richness (*Ar*), inbreeding coefficient (*F*_IS_), Garza–Williamson index (*G–W*) and *P*-values of Bayesian clustering analysis (for *K* = 1 and *K* = 2); mean ± SD

	Peak (1999)	Crash (2000)	Peak (2002)	Crash (2003)	Peak (2005)	Crash (2006)
*He*	0.866 ± 0.052	0.859 ± 0.058	0.865 ± 0.059	0.863 ± 0.053	0.859 ± 0.061	0.849 ± 0.076
*Ho*	0.826 ± 0.108	0.834 ± 0.094	0.857 ± 0.109	0.829 ± 0.077	0.835 ± 0.112	0.869 ± 0.090
*Ne1*	8050 (4740–9910)	5420 (4170–9910)	5210 (3010–9780)	5580 (6120–9800)	5080 (2790–9770)	4490 (5510–9770)
*A*	13.22	13.13	14.26	13.17	13.35	12.65
*Ar*	11.76 ± 3.75	11.66 ± 4.20	12.41 ± 4.97	11.74 ± 4.58	11.82 ± 4.83	11.64 ± 4.25
*F*_IS_	0.068 ± 0.112	0.051 ± 0.097	0.033 ± 0.101	0.062 ± 0.074	0.051 ± 0.118	0.001 ± 0.074
*G-W*	0.791 ± 0.185	0.779 ± 0.179	0.822 ± 0.146	0.801 ± 0.188	0.803 ± 0.174	0.795 ± 0.168
Bayesian clustering	*K* = 1, *P* = 0.3153	*K* = 1, P = 1.000	*K* = 1, *P* = 0.6401	*K* = 1, *P* = 0.0086	*K* = 1, *P* = 0.5529	*K* = 1, *P* = 0.4650
	*K* = 2, *P* = 0.6674	*K* = 2, P = 0.000	*K* = 2, *P* = 0.1977	*K* = 2, *P* = 0.9893	*K* = 2, P = 0.3760	*K* = 2, *P* = 0.2012

1 Median and 95% confidence interval.

Allelic richness (*Ar*) was estimated for each locus (Table A1) and study year (Table 1), and it ranged from 4.96 to 24.34 overall years. When comparing the peaks and crashes, we found the allelic richness to be only slightly (but significantly) lower at crashes (mean *Ar* = 11.68) when compared to peaks (mean *Ar* = 12.00) (Fig. 3b, randomization test, 10,000 permutations, *P* = 0.049).

The estimated pairwise *F*_ST_ values between study years varied between –0.0012 and 0.0104 (Table 2). Some of these values proved to be significant (randomization test with FDR; see Table 2), but there were no differences between the global *F*_ST_ values when we compared the crash (*F*_ST_ = 0.005) and peak (*F*_ST_ = 0.004) cycle phases (randomization test, *P* = 0.523). However, there was significant correlation (Mantel test, *r* = 0.4335, *P* = 0.033) between the genetic distance (*f*_ST_) and temporal distance (years) of the sample pairs, suggesting a slight change in the genetic structure of the populations during the study period.

**Table 2:** The pairwise *F*_ST_ values (below the diagonal) of the eight analyzed years of the cyclic bank vole population, *P*-values above the diagonal

	Peak (1999)	Crash (2000)	Peak (2002)	Crash (2003)	Peak (2005)	Crash (2006)
Peak (1999)		0.07000	0.02667*	0.01333*	0.00333*	0.00333*
Crash (2000)	0.0002		0.87000	0.08667	0.19333	0.00667*
Peak (2002)	0.0032	−0.0010		0.24000	0.06667	0.01000*
Crash (2003)	0.0019	0.0013	−0.0012		0.12000	0.01667
Peak (2005)	0.0040	0.0009	0.0053	0.0033		0.00333*
Crash (2006)	0.0104	0.0090	0.0083	0.0049	0.0102	

* *P* < 0.05.

diyabc was used to estimate effective population size and its changes over time. Direct estimates and logistic regression estimates gave in fact slightly stronger support (0.502, 0.514) to the model with constant population size compared to the one which allowed the population size to vary (0.498, 0.486), suggesting that even though the survey size of population decreased during the crash phases, the effective population size was much more constant (Table 1). In the model with constant population size, the posterior median of *Ne* was estimated to be 21,500 (95% confidence limits: 5680, 81,800). In the posterior distributions in the varying population size model, the median of the peak years (6113; SD = 1679) was not higher than the median of the crash years (5163; SD = 589; Mann–Whitney *U* = 4.0; *P* > 0.05). However, the confidence intervals for the estimates were so high that it is not easy to draw any definite conclusions on the relative magnitude of effective population sizes on peak and crash years. Still, it is quite clear that they remain relatively high during the crash periods.

In our analysis of population bottlenecks, we found no evidence for heterozygosity excess at any of the study years (Wilcoxon sign-rank test) and the allele frequency distributions were typically L-shaped, as expected under mutation-drift equilibrium for nonbottlenecked populations. The Garza–Williamson indices (Table 1) did not suggest that bottlenecks had occurred in any cycle phase. Across all sites and loci, the M-ratio ranged from 0.779 to 0.882 (Table 1) that, in each case, was higher than the critical value of 0.68 proposed by Garza and Williamson (2001).

### Spatial distribution, new alleles, and signals for selection

The Bayesian analysis of population structure (Pritchard et al. 2000; Falush et al. 2003) indicated the existence of a single panmictic population for the combined dataset (*K* = 1, *P* = 0.931). The other models (*K*≥ 2–8) were insufficient to explain the data (*P* = 0.069 for *K* = 2 and *P* < 0.0001 in other cases). When we analyzed the years separately, the most probable model varied between *K* = 1 and *K* = 2 (Table 1). The *P*-value for the most probable *K* varied between 0.4650 and 1.000, but there was no association between *K* and the cycle phase.

**Figure 4:**
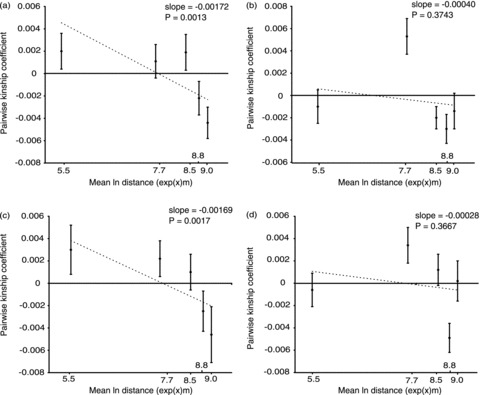
The spatial genetic structure expressed as kinship coefficient (mean ± SE) versus logarithmic distance between the individuals in the cyclic bank vole population. (a) Crash years, (b) peak years, (c) males, and (d) females.

The spatial autocorrelation analysis suggested local genetic structure within the population during low-density but not during the high-density phases (permutation test, 10,000 randomizations). The analysis of crash years showed that the negative regression slope (*b* = –0.0017) between the kinship coefficient and logarithmic distance between individuals was significant (*P* = 0.0013, Fig. 4a), suggesting restricted dispersal. On the contrary, the regression slope of peak years (*b* = –0.0004) was not significant (*P* = 0.3743, Fig. 4b). When we analyzed the sexes separately with all the study years combined, a significant population structure was evident among males (*b* = –0.0017, *P* = 0.0017, Fig. 4c), but not among females (*b* = –0.0005, *P* = 0.3667, Fig. 4d). The regression slopes for both females and males at peak years were not significant (females: *b* = 0.0010, *P* = 0.3209; males: *b* = 0.0010, *P* = 0.2065). The crash years were characterized by negative slopes, and the regression between kinship coefficient and logarithmic distance was significant among males (*b* = –0.0053, *P* = 0.0005) but not among females (*b* = –0.0014, *P* = 0.2299).

The mean number of novel (private) alleles per locus (Fig. 3c) was significantly higher in peak (mean = 3.391) than in crash (mean = 1.957) years (paired *t* test, *t* = 2.478, df = 22, *P* = 0.021), suggesting that the emerging alleles in peak phases are novel ones, instead of reappearing again after being lost during a crash phase.

The AMOVA revealed that three of the loci (Cg2F2, Cg1E6, and Cg12E6) showed deviation between the crash and peak years, but the *F*_ST_ values for all loci were low and there were no significant differences (after FDR correction) between the cycle phases. Moreover, 99.92% of the variation was within a cycle phase and only 0.08% among the cycle phases (1000 permutations, *P* = 0.314), which indicates that there are no large differences in the genetic composition between peak and crash phases of the population cycle.

## Discussion

We surveyed a population of bank voles for eight years—a period of three successive cycles of population density peaks and crashes. Our aim was to provide a new and comprehensive examination to the question whether the population's genetic diversity is maintained in spite of repetitive crashes in population size during the density cycle. We also wanted to evaluate possible mechanisms, such as spatial distribution and selection, acting to maintain genetic variability. In sum, we found no biologically significant differences in genetic variability between the crash and peak phases. Allelic richness was the only variable that showed marginal differences according to the cycle phase. Hence, we suggest that the high levels of genetic variability observed throughout the population cycle are (mainly) the consequence of (1) the effective population size being high even during the crash phases and (2) the appearance of new allelic combinations, most likely due to migration. On the other hand, we did observe a divergent spatial genetic pattern between crash and peak density phases, indicating differences in the spatial distribution of the individual genotypes during the opposing cycle phases.

The studies on the genetic diversity of cyclic rodent populations, the present study included, show high heterozygosity values in different phases of population cycle (Plante et al. 1989; Berthier et al. 2005, 2006; Ehrich and Jorde 2005; Redeker et al. 2006; Ehrich et al. 2009). Compared to heterozygosity, however, allelic richness reflects past fluctuations in population size more accurately (see e.g., Caballero and Rodriguez–Ramilo 2010). Allelic richness is a more sensitive estimator of short and severe genetic crashes than heterozygosity, since rare alleles are lost first during bottlenecks, which in turn has greater effect on allelic richness than on heterozygosity (Nei et al. 1975; Spencer et al. 2000). In the present study, allelic richness was temporarily lowered during the crash phases (Fig. 3b; Table 1). This was, to our knowledge, the first time that this kind of measurable reduction in genetic diversity has been demonstrated in a cyclical rodent population. However, we did not find a bottleneck signature, that is, heterozygosity excess, in the population after the crash phases. Heterozygosity excess is a commonly used measure of a population bottleneck that takes advantage of the fact that rare alleles are lost quickly during a bottleneck (Maruyama and Fuerst 1985) and the frequencies of remaining alleles change from their proportions prior to the bottleneck (Luikart and Cornuet 1998). Neither did the Garza–Williamson indices, which can detect bottlenecks despite low levels of migration (Swatdibong et al. 2010), suggest demographic bottleneck at any study year in our dataset. In addition, the population showed temporal genetic stability, since the *F*_ST_ values increased only slightly during the time separating the temporal samples (Table 2). Also the effective population size was estimated to be fairly constant and remained relatively high throughout the analyzed period (Table 1). Taken together, these data suggests that the genetic diversity of the population in crash phases is still high although slightly lower than in peak phases, and this marginal difference is quickly erased after every crash when new alleles enter into the population (Fig. 3c). Consequently, the population does not undergo severe genetic bottlenecks and the heterozygosity levels remain high and fairly constant over the whole analyzed period instead.

One possible explanation for the maintenance of genetic diversity in cyclic populations is the interplay of migration and genetic drift. The loss of genetic diversity that can be induced by drift within subpopulations during population crashes may be outweighed by the intensive migration between subpopulations, as proposed by Berthier et al. (2006), who analyzed a fossorial water vole (*Arvicola terrestris*) population for one increasing phase of the population cycle. When population density is low, the arrival of just a few immigrants that successfully reproduce can overcome genetic drift and balance out bottleneck effects (Keller et al. 2001). Ehrich and Jorde (2005) showed by simulating the strongly oscillating lemming-like dynamics that immigration can retard the loss of genetic diversity and the model has gained some experimental support (Berthier et al. 2006; but see also Berthier et al. 2005; Ims and Andreassen 2005). Our present long-term study shows repetitive and phase-dependent changes in the spatial distribution of the individuals and in the number of private alleles. During the peaks, the accumulation of new alleles (i.e., alleles not discovered before within the population) and the appearance of a homogenous population structure suggest higher migration rates and, consequently, increased gene flow within the population compared to the crash periods. The novel alleles probably constitute the source for various new allelic combinations and contribute to the overall heterozygosity of the population.

According to the above scenario, during crash phases the population might exhibit more patchy population structure and comprise larger number of reproductive clusters when compared to the peak phases. However, our Bayesian analysis did not indicate differences in number of breeding units between the peak and crash phases suggesting that genetic discontinuities, such as empty patches, do not appear within the studied bank vole population even during the crash phases. Despite the substantial crashes in the population size measured as the trapping index, the population apparently never reaches very low numbers and therefore a subpopulation structure with discrete breeding units does not form during the crashes. On the other hand, discrete breeding units may not form if the migration of individuals is high enough even during the crash phases (see Ims and Andreassen 2005). It should be noted here that our large-scale sampling of the data supports mainly landscape patterns. Future work may consider a more intensive small-scale sampling (i.e., multiple individuals per sampling site) to draw more specific conclusions about population's genetic structure.

An alternative way to maintain the high genetic diversity within a population is to avoid mating between relatives. The inbreeding avoidance can be achieved by different mechanisms such as dispersal or kin recognition, both of which have been documented in the microtine rodents (Bollinger et al. 1993; Kruczek 2007). Bank voles are energetic dispersers with dispersal distances extending up to 8.5 km (reviewed by Gliwicz and Ims 2000), and the dispersal of microtine rodents has been understood to be more frequent in higher than in lower population densities (reviewed by, e.g., Ishibashi et al. 1998, but see Ims and Andreassen 2005). Our present study indicates both low inbreeding within the population (*F*_IS_ ranging from 0.001 to 0.068, Table 1) and differences in the spacing behavior of individuals between the opposing cycle phases. During the peaks, the population's spatial genetic structure is more homogenous compared to crashes (Fig. 4a and b). This signals an increase in migration (or dispersal) when population size is large, which is also suggested by Berthier et al. (2006). Accession of new alleles into the population during the peak phases (Fig. 3b) can be a consequence of this activated movement of individuals. In microtine rodents, the dispersal occurs more frequently in males than in females (Bondrup-Nielsen and Karlsson 1985; Le Galliard et al. 2012). However, our results reveal that the females comprise more homogenous population in terms of genetic similarity compared to the males, whereas the males display some degree of isolation-by-distance (Fig. 4c and d). Moreover, during the crashes the population structure (isolation-by-distance) is originated by male individuals. This is somewhat ambiguous, since the bank vole females are known to exhibit territorial behavior and kin structure, which is promoted by the relatedness of the individuals (Mappes et al. 1995; Lambin and Yoccoz 1998). Whether this would be the case in the presently analyzed population as well is unfortunately not possible to answer with the current data, since the territoriality effect might act only at nearby localities, and our sampling strategy including only one adult individual per sampling location did not allow spatial genetic analysis at local level. In the common vole (*Microtus arvalis*), however, the long-distance dispersal is common also in females, although short-distance dispersal is male biased (Gauffre et al. 2009). The function of the short-distance dispersal is probably the inbreeding avoidance (Perrin and Goudet 2001), whereas the dispersal over long distances might function in colonizing new areas or in escaping crowding (Handley and Perrin 2007). Therefore, the genetic similarity within the female bank voles (Fig. 4d) could arise from the long-distance movements of the individuals during the peak phases. As far as spatial autocorrelation is concerned, a single correlogram may not reflect accurately the true nonrandom spatial genetic pattern. The significance of the autocorrelation largely depends on the extent of the genetic structure, the size of the distance class chosen and the associated number of samples per distance class (Peakall and Smouse 2006).

One mechanism for the maintenance of genetic diversity within populations is environmental heterogeneity (Roff 2002). This process, namely density-dependency, has been previously shown to operate in the bank vole (Mappes et al. 2008; but see also Gaines and Whittam 1980). The model of the linkage between selection on alternative life-history tactics and genetics was originally suggested by Chitty (1967) whose theory stated that the demography of small rodent populations was determined by the existence of opposing genetic morphs of individuals. We wanted to test whether one or more of the loci were linked to a particular cycle phase and would therefore signal for phase-dependent selection on that locus. Although three loci differed between the cycle phases according to the AMOVA, the deviations were not statistically significant. On the contrary, it seems that the alleles that are lost during the crash phases do not reappear, but are replaced by novel alleles instead. Nevertheless, we want to emphasize that this study only applies to the analyzed loci that were considered to be selectively neutral. Therefore, our results do not rule out the possibility of density-dependent selection to work on the vole population.

To conclude, our long-term study in the bank vole shows that severe cyclical population dynamics can have only minor effects on population's genetic diversity. The peak phases are characterized by both a change in spatial distribution of the individuals and a rapid accession of new alleles. Based on the present results, we propose that the constant and relative large effective population size, increased individual movement, and the consequential accumulation of new alleles during the peaks are a premise for the maintenance of high genetic diversity within cyclic rodent populations.

## Appendix

**Table A1:** Characteristics of the 23 studied microsatellite loci in the eight analyzed cycle phases of the bank vole population; expected heterozygosity (*He*), observed heterozygosity (*Ho*), number of alleles (*A*), and allelic richness (*Ar*)

Locus	Peak 1999 (*N* = 22)				Crash 2000 (*N* = 22)				Peak 2002 (*N* = 22)				Crash 2003 (*N* = 22)				Peak 2005 (*N* = 22)				Crash 2006 (*N* = 20)			
	*He*	*A*	*Ar*	*Ho*	*He*	*A*	*Ar*	*Ho*	*He*	*A*	*Ar*	*Ho*	*He*	*A*	*Ar*	*Ho*	*He*	*A*	*Ar*	*Ho*	*He*	*A*	*Ar*	*Ho*
Cg2D8	0.892	13	12.13	0.857	0.899	13	12.00	0.682	0.908	16	14.11	0.864	0.902	13	12.25	0.864	0.875	11	10.29	0.727	0.838	11	10.15	0.850
Cg2F2	0.836	10	9.28	0.773	0.840	11	10.12	0.857	0.874	13	11.72	0.864	0.850	10	9.59	0.810	0.837	10	8.89	0.955	0.878	13	11.92	0.950
Cg3F12	0.840	11	10.02	0.682	0.794	11	9.90	0.773	0.841	9	8.57	0.682	0.841	9	8.38	0.818	0.747	9	8.23	0.864	0.833	9	8.69	0.800
Cg3E12	0.870	14	12.20	0.818	0.863	11	10.22	0.909	0.800	10	9.33	0.682	0.792	9	8.52	0.818	0.805	7	6.91	0.818	0.809	10	9.16	0.800
Cg1F11	0.789	11	9.48	0.773	0.848	13	11.54	0.818	0.881	16	13.80	0.955	0.823	13	11.00	0.818	0.776	13	11.19	0.682	0.734	8	7.39	0.850
Cg5G6	0.885	11	10.16	0.955	0.836	11	9.75	0.773	0.853	9	8.65	0.857	0.846	8	7.66	0.818	0.884	10	9.83	0.909	0.890	12	11.48	0.900
Cg6D10	0.874	14	12.58	0.909	0.821	11	9.95	0.909	0.841	10	9.29	0.864	0.881	13	11.70	0.955	0.871	13	11.68	0.955	0.844	12	10.76	0.850
Cg8A5	0.907	16	14.25	0.636	0.913	19	16.4	0.818	0.925	21	17.48	0.864	0.910	19	15.98	0.864	0.917	17	14.89	0.909	0.924	17	15.64	0.900
Cg1E6	0.918	21	17.45	1.000	0.929	27	21.44	0.955	0.956	31	24.34	0.955	0.943	24	20.52	0.857	0.948	29	22.94	0.864	0.936	21	18.77	0.950
Cg17C9	0.902	14	12.90	0.909	0.890	13	11.79	0.909	0.899	14	12.71	0.818	0.905	14	12.77	0.864	0.855	13	11.21	0.682	0.894	12	11.61	0.950
Cg17A7	0.712	6	5.65	0.773	0.686	6	5.64	0.636	0.709	7	6.37	0.682	0.748	7	6.64	0.727	0.771	6	5.93	0.727	0.636	5	4.96	0.650
Cg16E2	0.893	13	11.95	0.773	0.861	11	10.00	1.000	0.843	10	9.10	0.909	0.830	9	9.00	0.875	0.849	10	9.45	0.905	0.850	11	10.29	0.900
Cg15F7	0.821	10	8.82	0.818	0.820	10	8.62	0.864	0.790	10	8.77	0.864	0.838	9	8.51	0.818	0.842	11	9.77	1.000	0.781	9	8.39	0.800
Cg13G2	0.827	9	8.36	0.773	0.855	11	9.80	0.810	0.861	12	10.92	0.762	0.827	9	8.23	0.762	0.836	9	8.60	0.810	0.848	10	9.49	0.900
Cg12H10	0.914	18	15.59	0.955	0.928	17	15.50	0.864	0.913	18	15.50	1.000	0.913	18	15.66	0.864	0.916	17	15.04	0.955	0.931	20	17.71	0.950
Cg10F6	0.869	9	8.69	0.955	0.858	9	8.52	0.857	0.861	10	9.11	0.909	0.847	9	8.58	0.818	0.866	9	8.87	0.857	0.836	10	9.19	0.950
Cg10D11	0.858	10	9.31	0.636	0.855	9	8.70	0.667	0.793	8	7.44	0.591	0.864	10	9.31	0.591	0.862	10	9.31	0.636	0.781	8	7.39	0.650
Cg12E6	0.927	20	16.97	0.955	0.929	21	18.10	0.905	0.940	23	19.61	0.955	0.939	23	19.46	0.909	0.947	25	21.13	0.905	0.924	19	17.14	0.950
Cg13B8	0.914	18	15.50	0.864	0.942	23	19.62	0.909	0.946	26	21.23	1.000	0.952	28	22.63	0.955	0.947	24	20.39	0.909	0.944	23	20.47	0.900
Cg13C12	0.866	13	12.01	0.818	0.871	13	11.83	0.909	0.868	14	12.04	0.909	0.830	12	10.63	0.773	0.883	16	13.84	0.909	0.853	13	11.89	0.900
Cg13F9	0.825	9	8.36	0.864	0.820	8	7.64	0.864	0.852	13	11.45	0.864	0.852	11	9.77	0.773	0.852	10	9.35	0.818	0.893	13	12.12	0.800
Cg17E9	0.822	8	7.45	0.636	0.789	6	5.73	0.773	0.809	7	6.45	0.955	0.796	7	6.59	0.818	0.744	6	5.86	0.591	0.744	6	5.84	0.842
Cg12B9	0.945	26	21.37	0.864	0.900	18	15.38	0.727	0.924	21	17.48	0.909	0.927	19	16.63	0.909	0.927	22	18.19	0.818	0.928	19	17.17	1.000
